# Correction: Accounting for Genetic Architecture Improves Sequence Based Genomic Prediction for a *Drosophila* Fitness Trait

**DOI:** 10.1371/journal.pone.0132980

**Published:** 2015-07-13

**Authors:** Ulrike Ober, Wen Huang, Michael Magwire, Martin Schlather, Henner Simianer, Trudy F. C. Mackay

## Notice of Republication

This article was republished on June 24, 2015, to replace an incorrectly published version. Please download this article again to view the correct version. The originally published, uncorrected article and the republished, corrected article are provided here for reference.

## Supporting Information

S1 File Originally published, uncorrected article. (PDF)

S2 File Republished, corrected article. (PDF)
