# Supplementary figures and images for: Accounting for Genetic Architecture Improves Sequence Based Genomic Prediction for a Drosophila Fitness Trait
Source: PLoS One. 2015 May 7;10(5):e0126880. doi: 10.1371/journal.pone.0126880 (PMC4423967; doi:10.1371/journal.pone.0126880)

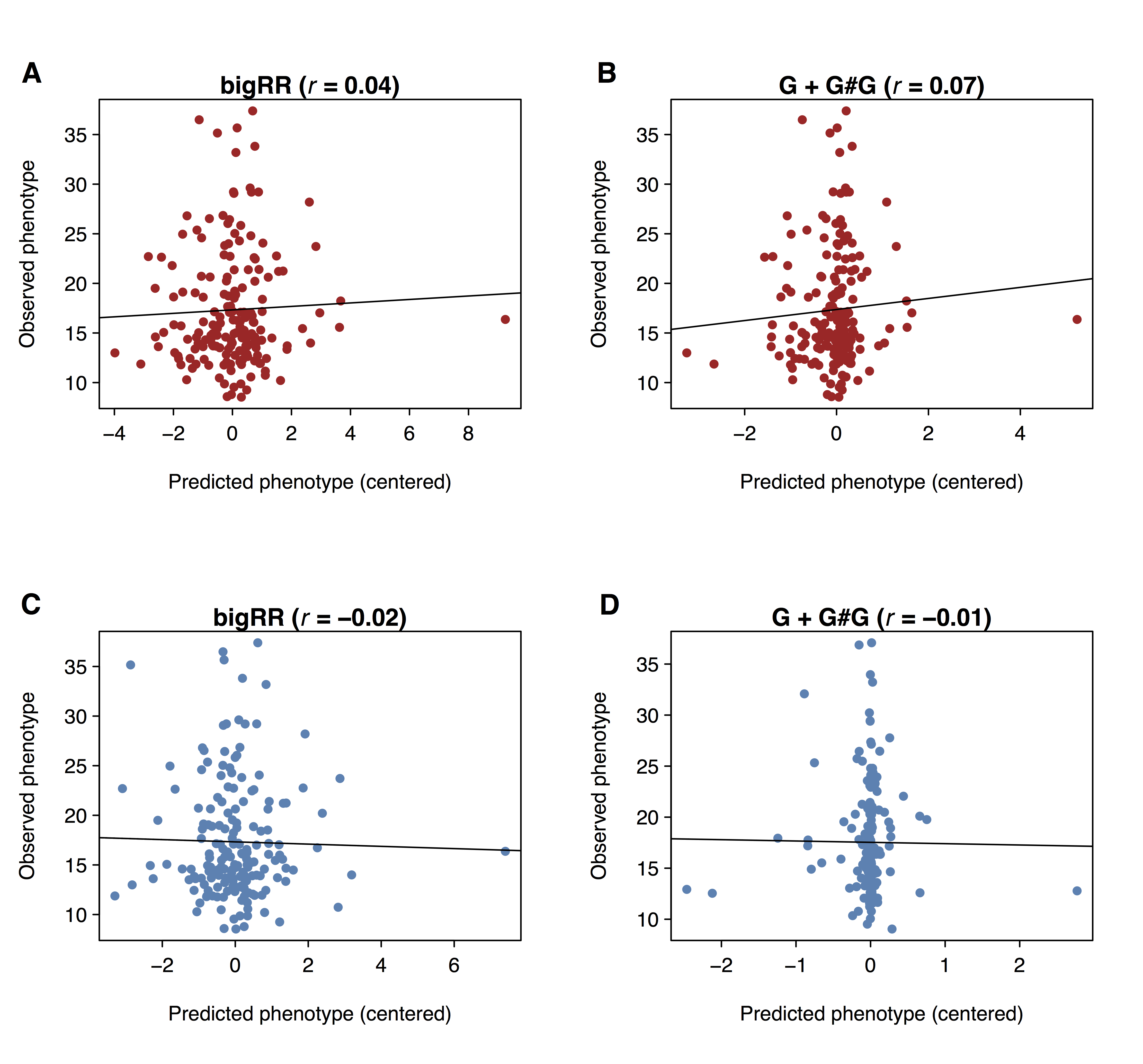

Supplement: S1 Fig — We performed LOOCV in females (A, B) and males (C, D) separately using a ridge regression approach implemented in the bigRR package [29] (A, females; C, males) and in the presence of genome-wide pair-wise epistatic variance (B, females; D, males). (TIF) [file pone.0126880.s001.tif]
