# Supplementary material for: Accounting for Genetic Architecture Improves Sequence Based Genomic Prediction for a Drosophila Fitness Trait
Source: PLoS One. 2015 May 7;10(5):e0126880. doi: 10.1371/journal.pone.0126880 (PMC4423967; doi:10.1371/journal.pone.0126880)
Supplement: S2 Table — (DOCX) [file pone.0126880.s003.docx]

**Table S1. Analysis of variance of chill coma recovery time**

df, degrees of freedom; MS, type III mean squares; F, F-ratio test, $\hat{\sigma}^{2}$**,** variance component; $\hat{H}^{2}$, broad sense heritability from individual data; $\hat{H}_{\bar{X}}^{2}$, broad sense heritability of line means.

| **Analysis** | **Source of Variation** | **df** | **MS** | **F** | ***P*-value** | $\hat{\sigma}^{2}$ **(SE)** | $\hat{H}^{2}$ **(SE)** | ${\hat{\boldsymbol{H}}}_{\bar{\boldsymbol{X}}}^{\boldsymbol{2}}$ **(SE)** |
| --- | --- | --- | --- | --- | --- | --- | --- | --- |
| Sexes | Sex (*S*) | 1 | 406.31 | 0.91 | 3.41E-01 | Fixed | 0.352 (0.04) | 0.879 (0.11) |
| Pooled | Line (*L*) | 175 | 5,857.64 | 13.04 | 1.07E-52 | 27.33 (3.17) |  |  |
|  | *S* × *L* | 175 | 448.97 | 1.81 | 1.40E-6 | 2.00 (0.51) |  |  |
|  | Replicate (*S* × *L*) | 361 | 248.44 | 4.98 | 1.32E-182 | 4.02 (0.37) |  |  |
|  | Error | 34,766 | 49.91 |  |  | 49.92 (0.38) |  |  |
| Females | Line (*L*) | 175 | 3501.40 | 17.08 | 2.20E-63 | 32.93 (3.75) | 0.373 (0.04) | 0.916 (0.10) |
|  | Replicate (*L*) | 181 | 204.88 | 3.70 | 8.97E-56 | 3.03 (0.44) |  |  |
|  | Error | 17,453 | 55.37 |  |  | 55.38 (0.59) |  |  |
| Males | Line (*L*) | 175 | 2,824.77 | 9.66 | 4.20E-44 | 25.67 (3.08) | 0.366 (0.04) | 0.836 (0.10) |
|  | Replicate (*L*) | 180 | 292.24 | 6.58 | 2.84E-141 | 5.05 (0.63) |  |  |
|  | Error | 17,313 | 44.41 |  |  | 44.41 (0.48) |  |  |
