# Supplementary material for: Accounting for Genetic Architecture Improves Sequence Based Genomic Prediction for a Drosophila Fitness Trait
Source: PLoS One. 2015 May 7;10(5):e0126880. doi: 10.1371/journal.pone.0126880 (PMC4423967; doi:10.1371/journal.pone.0126880)
Supplement: S4 Table — (DOCX) [file pone.0126880.s005.docx]

**Table S3.** **Variance components from models partitioning total genetic and additive genetic variance using the genomic relationship matrix.** $\hat{\sigma}^{2}$(CI) denotes the relevant variance component (confidence interval).

| **Analysis** | **Sex** | **Model** | **Source of Variation** | $\hat{\sigma}^{2}$**(CI)** |
| --- | --- | --- | --- | --- |
|  |  |  |  |  |
| **Individual Data** | **Female** | Total genetic variance | Total Genetic | 32.93 (3.75) |
|  |  |  | Replicate (Line) | 3.02 (0.44) |
|  |  |  | Error | 55.38 (0.59) |
|  |  |  |  |  |
|  |  | Non-additive | Non-additive Genetic | 18.50 (14.91) |
|  |  | + additive genetic | Additive Genetic | 14.36 (15.24) |
|  |  | variance | Replicate (Line) | 3.032 (0.44) |
|  |  |  | Error | 55.375 (0.59 |
|  |  |  |  |  |
|  |  |  |  |  |
|  | **Male** | Total genetic variance | Total Genetic | 25.67 (3.08) |
|  |  |  | Replicate (Line) | 5.04 (0.63) |
|  |  |  | Error | 44.41 (0.48) |
|  |  |  |  |  |
|  |  | Non-additive | Non-additive Genetic | 25.67 (3.08) |
|  |  | + additive genetic | Additive Genetic | 0 |
|  |  | variance | Replicate (Line) | 5.04 (0.63) |
|  |  |  | Error | 44.41 (0.48) |
|  |  |  |  |  |
|  |  |  |  |  |
| **Line Means** | **Female** |  | Additive Genetic | 14.04 (14.98) |
|  |  |  | Non-additive Genetic + error | 20.98 (14.68) |
|  |  |  |  |  |
|  |  |  |  |  |
|  | **Male** |  | Additive Genetic | 0 |
|  |  |  | Non-additive Genetic + error | 28.65 (3.06) |
|  |  |  |  |  |
