# Supplementary material for: Correction: Accounting for Genetic Architecture Improves Sequence Based Genomic Prediction for a Drosophila Fitness Trait
Source: PLoS One. 2015 Jul 13;10(7):e0132980. doi: 10.1371/journal.pone.0132980 (PMC4500411; doi:10.1371/journal.pone.0132980)
Supplement: S2 File — (PDF) [file pone.0132980.s002.pdf]

RESEARCH ARTICLE

Accounting for Genetic Architecture Improves Sequence Based Genomic Prediction for a *Drosophila* Fitness Trait

Ulrike Ober[1☯], Wen Huang[2☯], Michael Magwire[2¤], Martin Schlather[3], Henner Simianer[1], Trudy F. C. Mackay[2*]

1 Department of Animal Sciences, Animal Breeding and Genetics Group, Georg-August-Universität Göttingen, 37075, Göttingen, Germany, 2 Department of Biological Sciences, North Carolina State University, Raleigh, North Carolina, 27695–7614, United States of America, 3 Institute for Mathematics, University of Mannheim, 68131, Mannheim, Germany

☯ These authors contributed equally to this work.
¤ Current address: Syngenta, 3054 E. Cornwallis Road, Research Triangle Park, North Carolina, 27709, United States of America
* trudy_mackay@ncsu.edu

CrossMark
click for updates

OPEN ACCESS

Citation: Ober U, Huang W, Magwire M, Schlather M, Simianer H, Mackay TFC (2015) Accounting for Genetic Architecture Improves Sequence Based Genomic Prediction for a *Drosophila* Fitness Trait. PLoS ONE 10(5): e0126880. doi:10.1371/journal.pone.0126880

Editor: Suzannah Rutherford, Fred Hutchinson Cancer Research Center, UNITED STATES

Received: November 3, 2014

Accepted: April 8, 2015

Published: May 7, 2015

Copyright: © 2015 Ober et al. This is an open access article distributed under the terms of the Creative Commons Attribution License, which permits unrestricted use, distribution, and reproduction in any medium, provided the original author and source are credited.

Data Availability Statement: All raw data are available as S1 Table of this paper.

Funding: This research was funded by the German Federal Ministry of Education and Research (BMBF) within the AgroClustEr "Synbreed - Synergistic plant and animal breeding" (FKZ 0315528C) in association with the Deutsche Forschungsgemeinschaft (DFG) research training group "Scaling problems in statistics" (RTG 1644) to HS and by National Institutes of Health grant R01GM 45146 to TFCM. The funders had no role in study design, data

Abstract

The ability to predict quantitative trait phenotypes from molecular polymorphism data will revolutionize evolutionary biology, medicine and human biology, and animal and plant breeding. Efforts to map quantitative trait loci have yielded novel insights into the biology of quantitative traits, but the combination of individually significant quantitative trait loci typically has low predictive ability. Utilizing all segregating variants can give good predictive ability in plant and animal breeding populations, but gives little insight into trait biology. Here, we used the *Drosophila* Genetic Reference Panel to perform both a genome wide association analysis and genomic prediction for the fitness-related trait chill coma recovery time. We found substantial total genetic variation for chill coma recovery time, with a genetic architecture that differs between males and females, a small number of molecular variants with large main effects, and evidence for epistasis. Although the top additive variants explained 36% (17%) of the genetic variance among lines in females (males), the predictive ability using genomic best linear unbiased prediction and a relationship matrix using all common segregating variants was very low for females and zero for males. We hypothesized that the low predictive ability was due to the mismatch between the infinitesimal genetic architecture assumed by the genomic best linear unbiased prediction model and the true genetic architecture of chill coma recovery time. Indeed, we found that the predictive ability of the genomic best linear unbiased prediction model is markedly improved when we combine quantitative trait locus mapping with genomic prediction by only including the top variants associated with main and epistatic effects in the relationship matrix. This trait-associated prediction approach has the advantage that it yields biologically interpretable prediction models.

collection and analysis, decision to publish, or preparation of the manuscript.

Competing Interests: The authors have declared that no competing interests exist.

Introduction

The ability to accurately predict phenotypes for quantitative traits from genome wide polymorphism data will revolutionize evolutionary biology, medicine and human biology, as well as breeding of agriculturally important plant and animal species. The most commonly used experimental approach to dissect the genotype-phenotype map has been to identify individual quantitative trait loci (QTLs) by linkage to, or association with, segregating molecular markers in mapping populations [1]. These studies evaluate the null hypothesis that variants are not associated with differences in trait means, and rejection of the null hypothesis gives biological insight into genes and genetic networks affecting naturally segregating quantitative variation. Genome wide association (GWA) mapping studies for quantitative traits and complex diseases in humans have identified over 2,000 novel variants [2]. However, the effects of individual variants are small, and collectively they explain only a small fraction of the total genetic and phenotypic variation for each trait, a phenomenon termed 'missing heritability' [3].

A second approach computes genome-based relationship matrices among individuals in a population based on all genotyped segregating variants, and uses this to estimate the fraction of additive genetic variance explained by the variants [4, 5]. When applied to human quantitative traits and complex diseases, this method explains a much greater fraction of the total heritability than single marker analyses [6, 7]. Importantly, when a statistical model is developed in a population for which individuals have been both genotyped for molecular markers and phenotyped for a quantitative trait, the model can be used to predict phenotypes for an independent sample of individuals from the same population with genotype information only (genomic prediction). Genomic prediction methods have been extended to predict genomic breeding values for ranking selection candidates in animal and plant breeding programs (genomic selection) [8–12]. Genomic selection can massively increase genetic progress and is currently widely utilized in applied breeding programs.

With the advent of next generation sequencing technologies, we can now perform genome wide association mapping and genomic prediction on populations of individuals with complete genome sequences. This scenario differs from GWA and genomic prediction analyses in which only a subset of segregating variants are genotyped in that the causal variants are themselves included in the list of polymorphic variants. If the true genetic architecture of a trait differs from the additive, highly polygenic model typically assumed in genomic prediction (GBLUP, genomic best linear unbiased prediction) [13, 14], combining QTL mapping with genomic prediction may improve prediction accuracy and yield biologically relevant prediction models. Indeed, genomic prediction methods that incorporate epistatic or non-additive effects typically outperform their counterparts with only additive effects [15, 16].

The *Drosophila melanogaster* Genetic Reference Panel (DGRP) consists of 205 sequenced inbred lines derived from the Raleigh, NC population [17, 18]. Here, we report GWA and genomic prediction analyses for time to recover from a chill-induced coma, a component of fitness in *Drosophila* and other insects [19, 20]. We find substantial total genetic variation for chill coma recovery time, with a genetic architecture that differs between males and females and includes alleles with large additive effects as well evidence for epistasis [21]. Genomic predictive ability for chill coma recovery time is very low when based on a genomic relationship matrix including all markers, but is markedly improved when the relationship matrix only includes variants associated with main and epistatic effects on the trait.

Results

Quantitative genetics of chill coma recovery time

We assessed time to recovery from a chill-induced coma for 176 of the 205 DGRP lines (S1 Table) with Illumina sequence data [17, 18]. We find significant genetic variation ($P = 1.07 \times 10^{-52}$ for the between line variance and $P = 1.40 \times 10^{-6}$ for the sex by line interaction variance) for chill coma recovery time (Fig 1), with a broad sense heritability (± SE) of $\hat{H}^2 = 0.35$ (± 0.04) (S2 Table), similar to that previously reported for 157 DGRP lines [17]. The genetic correlation between chill coma recovery time in males and females is high ($\hat{r}_{MF} = 0.93$). However, given the significant sex by line interaction variance (S2 Table), we considered males and females separately in subsequent analyses.

Genetic architecture of chill coma recovery time

Our previous GWA analysis of chill coma recovery time used single nucleotide polymorphisms (SNPs) for 157 Freeze 1.0 DGRP lines [17]. Sequences of these lines were obtained using both 454 and Illumina technology. Illumina sequences are now available for all 205 Freeze 2.0 DGRP lines, which have been genotyped for SNPs as well as small and large insertion/deletion (indel) variants and other non-SNP variants [18]. We performed a GWA analysis for chill coma recovery time based on line means, using a mixed model to account for relatedness for 176 DGRP lines with Freeze 2 genotypes of 1,868,905 common (minor allele frequency ≥ 0.05) bi-allelic variants meeting quality control metrics. The broad sense heritabilities of line means (± SE) are $\hat{H}^2 = 0.92$ (± 0.10) in females and $\hat{H}^2 = 0.84$ (± 0.10) in males (S2 Table). The increase over individual-based heritability estimates is because the line means are estimated with much greater precision given that we measured approximately 100 individuals per sex and line.

At a nominal $P < 10^{-5}$ threshold, we found 68 variants in or near 44 genes associated with female chill coma recovery time and 68 variants in or near 42 genes associated with male chill coma recovery time (S3 Table). A total of 26 genes were male-specific, 28 were female-specific, and 16 were common to both sexes (although the variants associated with males and females for the common genes were not necessarily the same). Three SNPs in females (*2L*_3753356_SNP in the 3'UTR of *CG10019*, *2L*_4588764_SNP in the first intron of *dumpy* (*dp*), and *X*_18394766_SNP, a non-synonymous polymorphism in *Rad51D*) and one SNP in males (*2L*_4513330_SNP in the first intron of *dp*) had large effects and were genome-wide significant at a Bonferroni-corrected 5% significance threshold ($P < 2.68 \times 10^{-8}$; S3 Table). These few significant additive SNPs explained 36% and 17% of the genetic variation among lines in females and males, respectively. Previously, we inferred widespread epistasis for female chill coma recovery time from the failure of associations with common SNPs to replicate between the Freeze 1.0 DGRP lines and an extreme-QTL GWA analysis of a large advanced intercross population derived from 40 DGRP lines, despite adequate power [21]. We therefore infer that the genetic architecture of chill coma recovery time is sex-specific, with a small number of SNPs with large main effects and variants with smaller additive effects and epistatic interactions that account for the remaining genetic variance in each sex.

Genomic prediction for chill coma recovery time using genome wide variants

We constructed genomic relationship matrices [5] from all of the 3,742,106 SNPs and 437,096 indels that were polymorphic in the 176 DGRP lines, as well as for the subset of 1,868,905 common variants (MAF ≥ 0.05). We used genomic best linear unbiased prediction (GBLUP) to

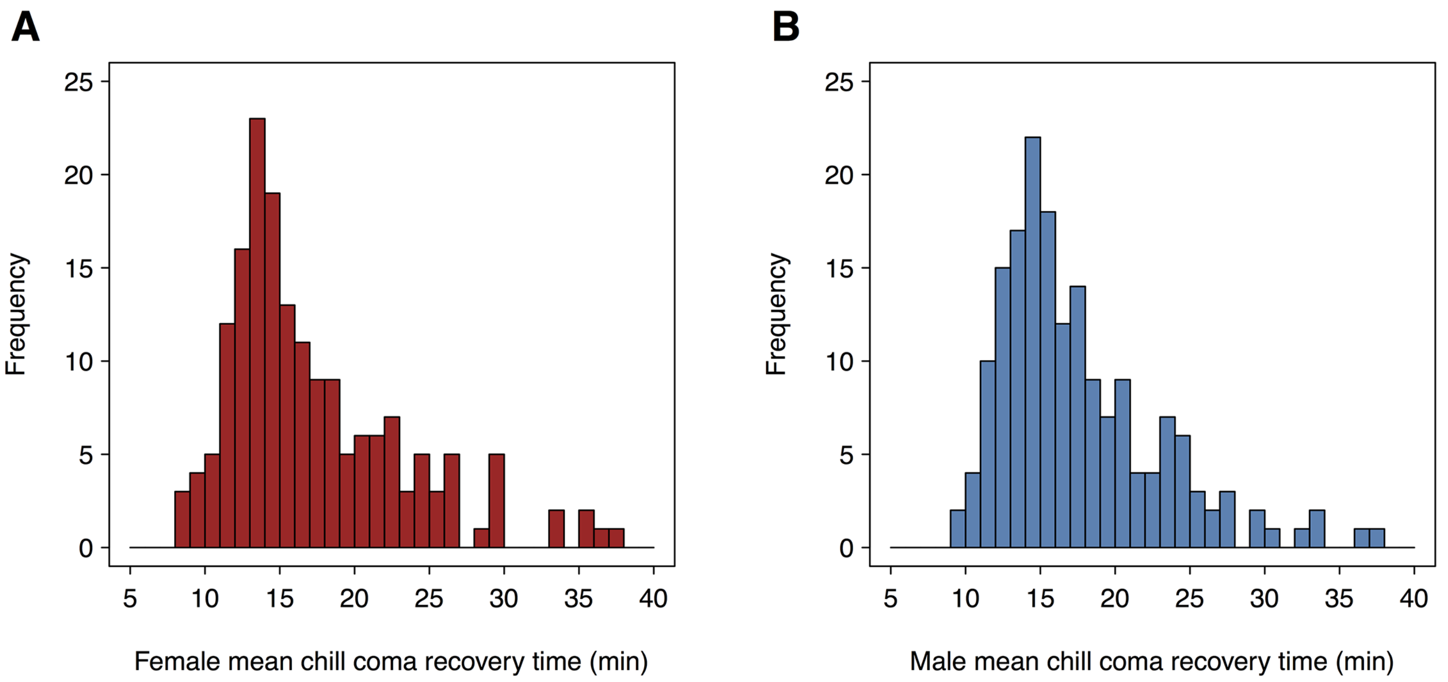

Fig 1. Distribution of chill coma recovery time among 176 DGRP lines. The histograms depict the distribution of line means for (**A**) females and (**B**) males for chill coma recovery time.

doi:10.1371/journal.pone.0126880.g001

predict mean chill coma recovery phenotypes for these lines, as described previously for starvation resistance and startle response [13].

We used 100 replicates of 5-fold cross-validation (CV) to assess the average correlation (r) between predicted genetic values using all common variants and observed phenotypes, for each sex separately. Surprisingly, we found that genomic-based predictive ability was very low ($r = 0.08$) in females, and in males, the estimate of additive genetic variance from the genomic relationship matrix was zero, leading to $r = 0$. Similar results ($r = 0.08$ in females and $r = 0$ in males) were obtained using all variants, suggesting that the low predictive ability of genomic prediction was not due to the omission of rare variants (Fig 2). The distributions of chill coma recovery times are not normal, and have a pronounced minor peak for longer recovery time in both sexes (Fig 1). The low predictive ability of genomic prediction is not, however, attributable to the non-normal distributions, as Box-Cox transformed data show the same pattern of low predictive abilities ($r = 0.10$ in females and $r = 0$ in males using common or all variants, Fig 2). This result is in contrast to previous analyses, where predictive ability for starvation resistance and startle response was 0.24 and 0.23, respectively [13].

The low predictive ability of genomic prediction could be due to low additive genetic variance for chill coma recovery time, despite appreciable total genetic variance. To assess this, we estimated the additive genetic variance for chill coma recovery using the genomic relationship matrix derived from common variants, both for individual data and for line means. Indeed, the estimate of additive genetic variance is zero for males and not significantly different from zero in females in both analyses (S4 Table). In fact, the likelihood profile for the additive genetic variance in males was flat near the origin and thus the maximum likelihood estimate was zero.

The observation that estimates of additive genetic variance using the genome-wide relationship matrix are not significantly different from zero is puzzling given the results from the GWA analyses. As noted above, we estimated that the top GWA hits accounted for 36% and

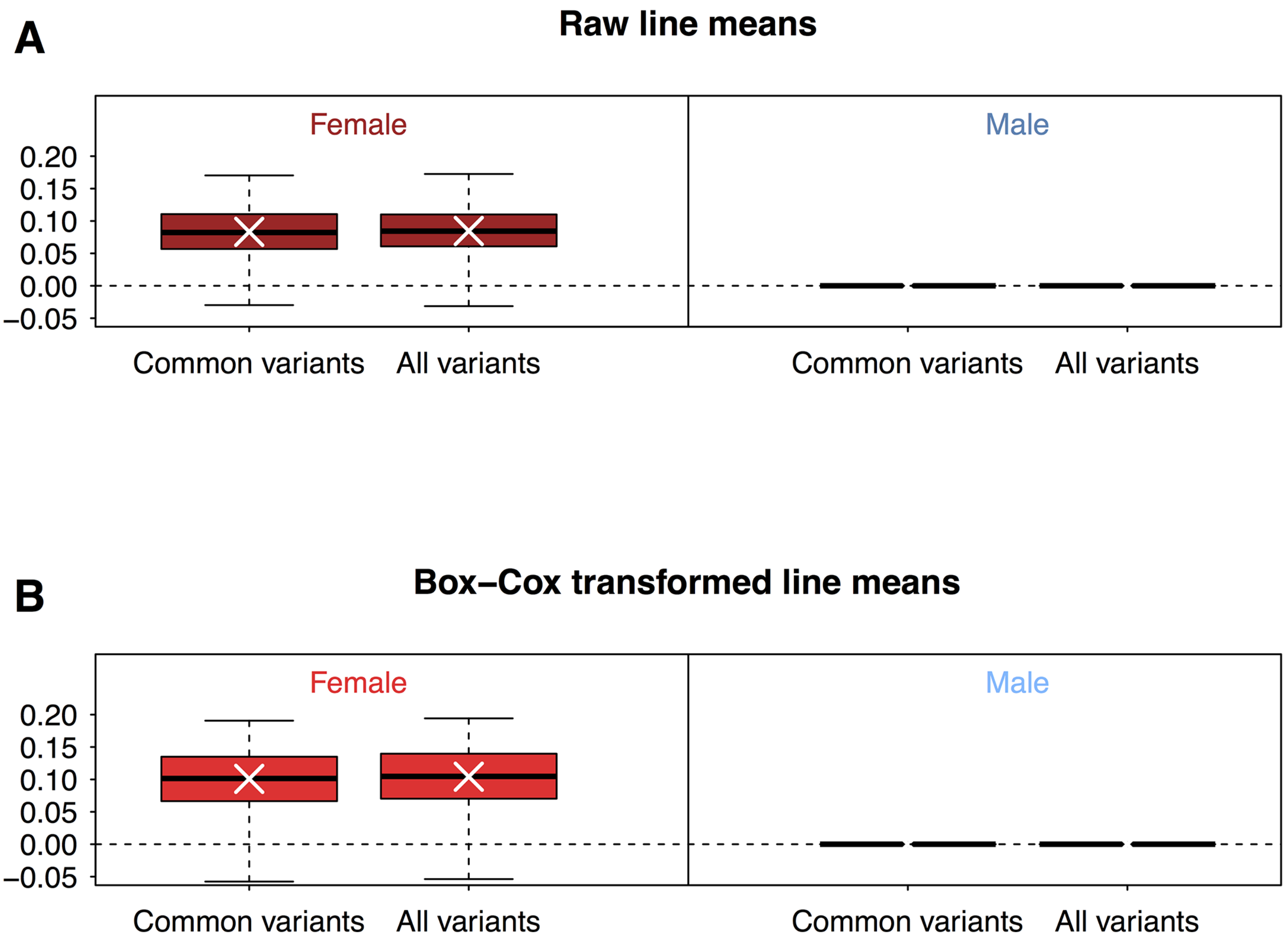

Fig 2. Prediction accuracy using common or all variants with raw line means or Box-Cox transformed line means. Prediction accuracy of GBLUP for 100 replicates of 5-fold cross-validation (CV) are plotted as box plots, for females and males separately. We performed the analysis using either raw line means (**A**) or line means transformed by Box-Cox transformation (**B**).

doi:10.1371/journal.pone.0126880.g002

17% of the variance among lines in females and males, respectively. There are several possible technical explanations for the discrepancy between the additive genetic variance expected to be contributed by variants detected in our GWA analysis and the non-significant or zero estimates of additive variance from the genome wide relationship matrix. First, the sample size of 176 lines is not large and may produce unstable estimates. However, the sample size was even smaller for starvation resistance and startle response in our earlier study in which the GBLUP estimates of additive genetic variance were reasonably accurate from a strictly additive model [13]. Second, the GBLUP model assumes a highly polygenic genetic architecture such that the effects of all variants are strictly additive, and normally distributed with equal variance. Departures of the true genetic architecture from these model assumptions, such as variants with large effects, non-additive genetic variance, or both, as inferred for chill coma recovery time, could thus lead to low GBLUP estimates of additive genetic variance. Further, the genomic variance estimated from the relationship matrix is not necessarily identical to the true genetic variance of the trait [22], although differences should be minor if the genomic relationship is constructed from sequence data assumed to harbor all causal variants, as in the present case.

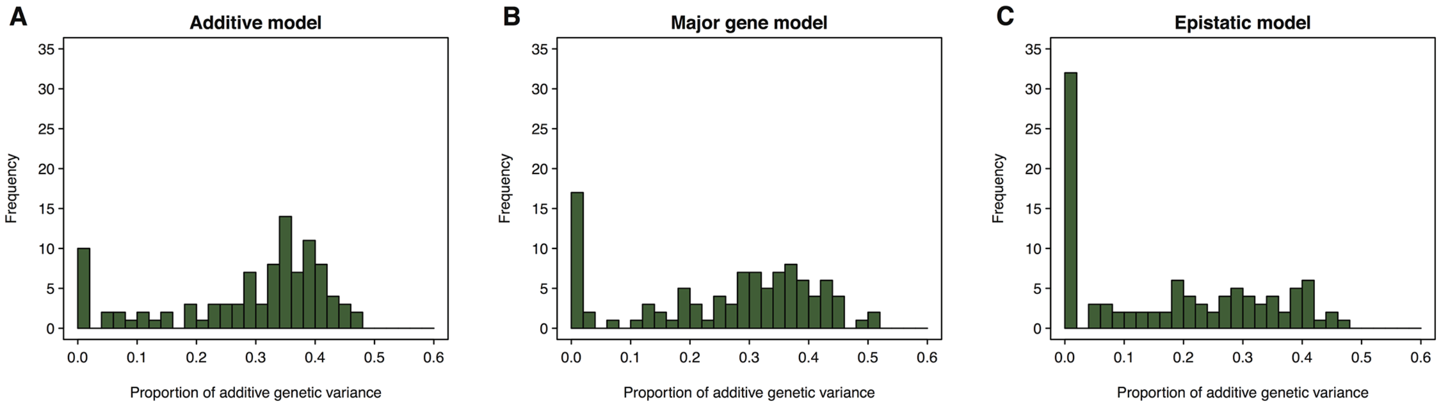

Fig 3. Genetic architecture affects estimated additive genetic variance. Different genetic architectures were simulated using DGRP genotypes for an additive model (**A**), a major gene model (**B**) or an epistatic model (**C**). Broad sense heritability was assumed to be 37%, the same as the observed chill coma recovery time. A total of 100 simulations were performed and the additive genetic variance (expressed as the proportion of additive genetic variance of total variance) was estimated and summarized in histograms.

doi:10.1371/journal.pone.0126880.g003

We assessed the effects of genetic architecture on estimates of additive genetic variance from the genomic relationship matrix by simulating phenotypes for the DGRP genotypes with different genetic architectures. We considered an additive model consisting of 100 QTLs each explaining 1% of the total genetic variance, a major gene model where one QTL explains all of the genetic variance, and an epistatic model where each of 50 pairs of interactions explains 2% of the total genetic variance. We simulated the phenotypic data such that the broad sense heritability is 37% (*i.e.*, the same as for chill coma) and estimated the additive genetic variance using a mixed model. We performed 100 replicate under each scenario. While the additive model occasionally led to low estimates of additive genetic variance, the major gene model did so slightly more frequently and the epistatic model substantially more frequently (Fig 3). Therefore, we conclude that departures from the genetic architecture assumed by the GBLUP model can cause a substantial underestimation of the additive genetic variance and hence reduce predictive ability of the model.

Incorporating genetic architecture improves genomic prediction

Because the underlying genetic architecture affects the amount of estimated additive genetic variance, which is the variance component accessible by GBLUP prediction, we assessed whether genomic prediction could be improved by incorporating additive and/or epistatic trait-associated variants. We used leave-one-out cross-validation (LOOCV) in these analyses to maximize sample size in the training set. In each of the 176 LOOCV iterations, one line was left out and the remaining 175 lines were used to carry out a GWAS for single variants and pair-wise interactions between variants. We then selected the top trait-associated additive variants and/or epistatic pairs with $P < 10^{-X}$ to construct the genomic relationship matrix and predict the phenotype of the remaining line. We computed the predictive ability as the correlation between the vector of estimated genetic values and the vector of observed line means, and varied X to arrive at an optimal threshold (Fig 4). Using all common variants, we again found that GBLUP had low predictive ability in females (r = 0.07) and none in males (Fig 5). However, the maximum predictive ability increased to 0.40 in males and 0.43 in females when only the top SNPs were used to construct the relationship matrix (Fig 4 and Fig 5). Using top epistatic variants to build a pairwise epistatic genomic relationship matrix based on a modified approach of Astle and Balding [23] also resulted in an improvement in predictive ability, to 0.35 in males

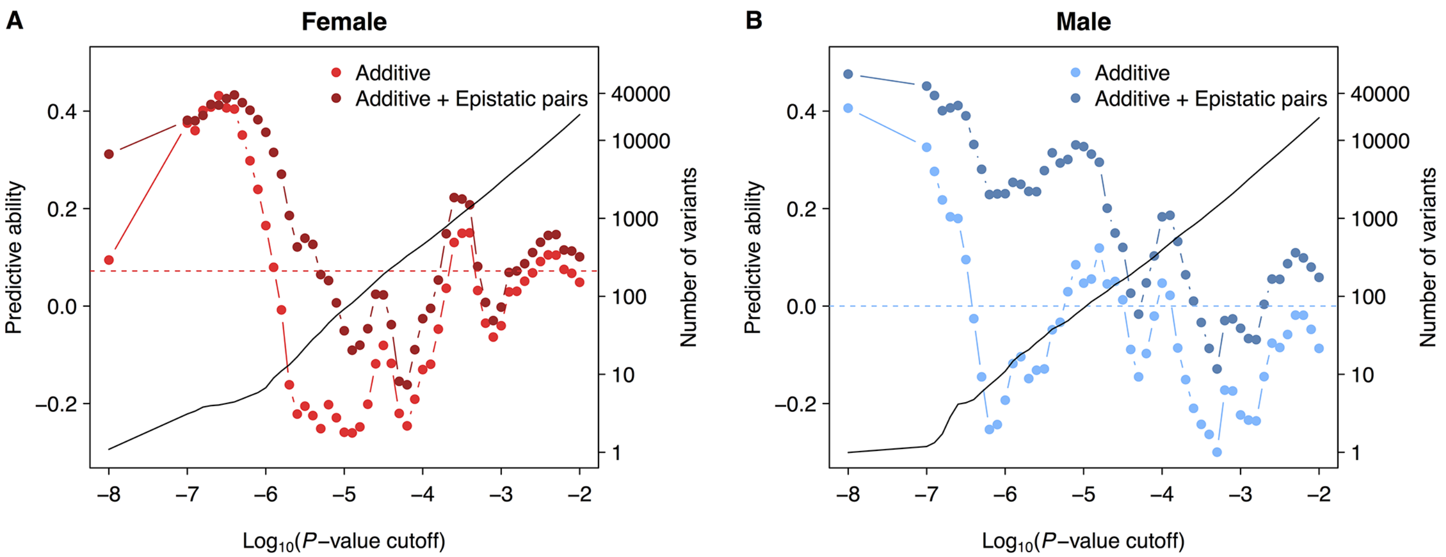

Fig 4. Trait-associated GBLUP. We performed LOOCV in females (**A**) and males (**B**) separately. In each of the 176 folds, the top GWAS associations and/or epistatic interactions in the training set were used to build the genomic relationship matrix and make prediction of the validation line. Accuracy of prediction (left *y*-axis, correlation between predicted and observed phenotypes) is plotted against the *P*-value threshold for the additive model and additive + epistatic model. For the additive + epistatic model, the epistatic pairs (on average the top 30 pairs in females and the top 3,232 pairs in males) that achieved the highest prediction accuracy in an epistatic only model was used, while the threshold for GWAS association was varied. The black line indicates the number of variants (right *y*-axis) significant in GWAS at varying thresholds.

doi:10.1371/journal.pone.0126880.g004

and 0.32 in females (Fig 5). Finally, incorporating both top additive variants and top epistatic variants (MAF > 0.15) improved the predictive ability in males to 0.48, but did not improve predictive ability in females (0.43) beyond that achieved by the additive model alone (Fig 5). In this combined model, we used the top epistatic variants leading to the best predictive ability in the epistatic LOOCV and varied the *P*-value threshold for the additive variants to optimize the predictive ability. Interestingly, in the additive only model, the highest predictive ability in males was achieved when a single variant was included (Fig 4), which coincided with the top association signal in males (*2L*_4513330, S3 Table).

Discussion

The sequenced inbred lines of the DGRP provide a unique opportunity to dissect the genetic architecture of *Drosophila* quantitative traits. Here, we performed GWA analyses for a component of fitness, time to recover from a chill-induced coma. The broad sense heritability of chill coma recovery time in the DGRP is moderately high (~0.40), and sex-specific. In contrast to results from most GWA analyses in human populations, in which the top GWA variants have small effects and collectively explain only a small fraction of the total heritability [2, 3, 24], we found that a small number of sex-specific SNPs with large marginal effects explain a substantial fraction of the variation in chill coma recovery time in the DGRP. These variants are common and are not in long-range linkage disequilibrium with other genomic variants, which can occur for variants with MAF < 0.05 due to the small size of the DGRP. In addition to large additive effects, our previous study implicated substantial epistasis for chill coma recovery time [21].

The complicated genetic architecture of chill coma recovery time is not unexpected for a fitness-related trait. Fisher's fundamental theorem of natural selection predicts reduced levels of additive genetic variation for such traits, with residual genetic variance attributable to additive and dominance variance from deleterious alleles maintained at low frequencies by mutation-selection balance and balancing selection of alleles at intermediate frequencies, and epistatic

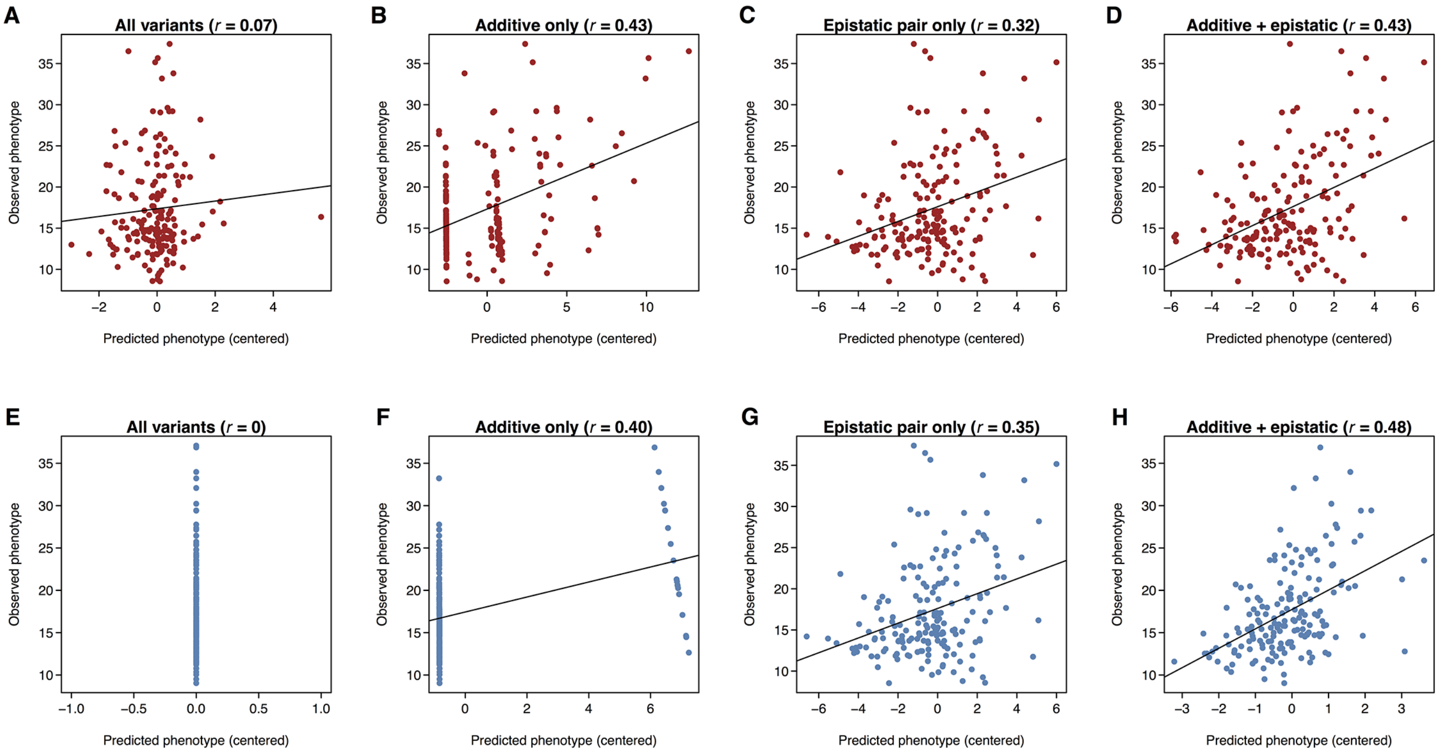

Fig 5. Accounting for genetic architecture improves genomic prediction. Scatter plots showing the predicted phenotypes and observed phenotypes for females (**A-D**) and males (**E-H**) under different GBLUP models. Each panel represents a model indicated by the text above the plot with the prediction accuracy in the parenthesis. Each point in the scatter plots represents one fold of LOOCV under the indicated model.

doi:10.1371/journal.pone.0126880.g005

variance from gene-gene interactions [25]. Our GWA analysis did not evaluate the effects of low frequency alleles (MAF < 0.05). However, the additive effects were predominantly such that the carriers of rarer alleles took longer to recover from a chill-induced coma (S3 Table). To the extent that allele frequency is a proxy for fitness, this suggests that these alleles are deleterious with respect to chill coma recovery, and that they may be under a form of balancing selection. Possibilities include overdominance for chill coma recovery time or a beneficial effect on another fitness trait.

We computed genetic relationship matrices for all common variants and used GBLUP and cross-validation to assess predictive ability for chill coma recovery time. GBLUP is only one of many methods utilized in genomic prediction [26–29]. Simulation studies show that methods tailored to specific known underlying genetic architectures, in particular Bayes B [4], give improved predictive abilities over GBLUP when the genetic architecture consists of a few, additive loci with large effects [26–28]. However, Bayesian methods are very sensitive to the assumed prior distribution of effects, which are unknown *a priori* [29]. Alternatively, a recently proposed generalized ridge regression method estimated variant-specific shrinkage parameters from the data without the need for prior distributions [30]. However, this method made predictions that were comparable to GBLUP (S1 Fig). GBLUP is not only easy to implement but gives equivalent (and high) prediction accuracies when compared to other methods when applied to plant and animal breeding populations [14, 26–28], in which the relationship between individuals in the training and test populations is high. In human populations, however, in which the individuals in the training and test populations are not related, GBLUP

prediction accuracy is not high [14, 31], suggesting that models incorporating genetic architecture could improve predictive ability.

When genetic architecture was not included in the model, the predictive ability we obtained for chill coma recovery time using GBLUP was very low for females and zero for males, due to estimates of narrow sense heritability that are not significantly different from zero in females and equal to zero in males. We hypothesized that this low predictive ability is due to violations of the additive infinitesimal model assumed by GBLUP that is inconsistent with the true genetic architecture of chill coma recovery time. This idea was supported by our simulation study (Fig 3). Our GWA analyses and previous results suggest that the true architecture of chill coma recovery includes a few large effect additive loci coupled with additional epistatic interactions. The low predictive ability from the mismatch between the true and assumed genetic architecture is further exacerbated by the low average genomic relatedness of the DGRP lines [13, 18] and rapid decline of LD with physical distance in the DGRP [17,8]. Whether the violation of the infinitesimal model was due to major genes, non-additivity or both cannot be definitively answered with the available data.

The GBLUP model utilizes the average genomic relationship over all loci between individuals (or lines, in the case of the DGRP), and is the same for all traits measured in the population [32]. Furthermore, although epistasis for chill coma appears to be widespread [21], inclusion of a second variance component accounting for genome-wide pair-wise interactions among all considered variants did not improve prediction (S1 Fig). This is expected since most genetic variance is additive even if the gene action is epistatic, unless allele frequencies are intermediate [33, 34]. However, if only a few loci or interactions between them affect the trait, using the entire genomic relationship matrix and/or genome-wide interactions essentially adds noise unless the average genomic relatedness matches the genomic relatedness at causal loci, leading to reduced prediction accuracy [14, 32]. Indeed, our implementation of GBLUP that takes account of trait-associated QTLs [32], including epistasis and major additive effects, dramatically improved predictive ability.

The genomic prediction was greatly improved when adding SNPs or SNP combinations selected based on GWA analyses in each fold of the LOOCVs. Although this study was underpowered to detect epistatic interactions globally due to its small sample size, this does not mean that there is no epistasis among the top hits in the pair-wise epistasis tests. The improvement of prediction accuracy is likely a result of enriching for true causal variants among the list of variants used to construct the genetic covariance matrix. In addition to improved predictive ability, the trait-associated GBLUP approach affords the opportunity to evaluate the stability of particular additive variants and pairwise interactions by enumerating the number of times each enters the training model in the different folds of the LOOCV (S5 Table and S6 Table). Not surprisingly, the top additive variants in the GWA analysis were recapitulated among the variants entering the model in all or most of the folds of the LOOCV (S5 Table). However, there were relatively few pairwise interactions repeatedly entering the models (S6 Table); these interactions have the highest priority for future functional tests.

Epistasis is a major feature of the genetic architecture of time to recover from a chill-induced coma in *Drosophila* [21]. To what extent does this phenomenon apply to other quantitative traits in *Drosophila*, as well as to quantitative traits in other species? There is substantial evidence for epistatic interactions between QTLs in *Drosophila* and mice [1, 21, 24, 34], chickens [35], *Arabidopsis* [36] and yeast [37, 38]. Epistatic effects can be as large as main QTL effects, and can occur in opposite directions between different pairs of interacting loci and between loci without significant main effects on the trait. Epistasis can also occur between closely linked QTLs [36–38] and even between polymorphisms in a single gene [39]. Therefore, epistasis appears to be a common feature of the genetic architecture of a wide range of quantitative traits

in genetic model organisms, and hence, by extension, in other less genetically tractable species as well.

The implications of pervasive epistasis are profound. Complex networks of genetic interactions provide the genetic basis of canalization (hidden genetic variation) [40]. Widespread and complex epistasis could lead to population-specific genetic architecture, since the effect of a given allele will be conditional on the presence of interacting alleles, which typically vary in frequency between populations [21, 34, 41]. This leads to non-replication of estimates of main effects in different populations and to population specific responses to artificial selection. Epistasis may be in part responsible for the phenomenon of missing heritability [3] in human complex traits and diseases, since estimates of effects of single markers are biased when epistasis exists but is not accounted for [34, 42]. In this regard epistasis provides a reason for the apparent additive and close to infinitesimal genetic architecture of many quantitative traits: additivity is an emergent property of underlying epistatic gene action [34]. We know that developmental, neural, transcriptional, metabolic and biochemical networks are highly dynamic, interconnected and nonlinear [43]. Identifying epistatic genetic interaction networks will greatly inform our understanding of how molecular interactions affect variation in organismal quantitative traits [34, 44], as well as improve genomic prediction.

Materials and Methods

Drosophila phenotypic data

We quantified chill coma recovery by transferring (without anesthesia) three to seven day-old flies to empty vials, and placing them on ice for three hours. We transferred the flies to room temperature, and recorded the time it took for each individual to right itself and stand on its legs [45]. We obtained two replicate measurements per sex and line, with 50 flies per replicate, for each of 177 DGRP lines. One line, DGRP_879, had a chill coma recovery phenotype two standard deviations from the mean, and was excluded from all analyses.

Heritability, genetic correlation, and variance components

To estimate broad sense heritability, we fitted the mixed model $Y = \mu + S + L + S{\times}L + R(S{\times}L) + \varepsilon$ to the individual level data. Y is phenotype, μ is the overall mean, S is the fixed effect of sex, L is the random effect of line, R is the random effect of replicate, and ε is the residual. We also fitted reduced models separately for males and females. We estimated the broad sense heritability for the full model as $\hat{H}^2 = \frac{\hat{\sigma}^2_L + \hat{\sigma}^2_{SL}}{\hat{\sigma}^2_L + \hat{\sigma}^2_{SL} + \hat{\sigma}^2_E}$, where $\hat{\sigma}^2_L$, $\hat{\sigma}^2_{SL}$ and $\hat{\sigma}^2_E$ are, respectively, the estimated variance components for the line, sex by line and residual terms. For the reduced models, our estimates of broad sense heritability were $\hat{H}^2 = \frac{\hat{\sigma}^2_{LM}}{\hat{\sigma}^2_{LM} + \hat{\sigma}^2_{EM}}$ for males and $\hat{H}^2 = \frac{\hat{\sigma}^2_{LF}}{\hat{\sigma}^2_{LF} + \hat{\sigma}^2_{EF}}$ for females, where the subscripts M and F refer to the sex-specific among- and within-line variance components. We estimated the genetic correlation between males and females as $\hat{r}_{MF} = \frac{\hat{\sigma}^2_L}{\hat{\sigma}^2_L + \hat{\sigma}^2_{SL}}$. We estimated heritabilities of line means from the individual data as $\hat{H}^2 = \frac{\hat{\sigma}^2_{LM}}{\hat{\sigma}^2_{LM} + \hat{\sigma}^2_{RM}}$ and $\hat{H}^2 = \frac{\hat{\sigma}^2_{LF}}{\hat{\sigma}^2_{LF} + \hat{\sigma}^2_{RF}}$ for males and females, respectively, where $\hat{\sigma}^2_R$ is the between replicate variance component. To partition total genetic variance into additive and non-additive components, we fitted a mixed model $\mathbf{y} = \mathbf{W}\mu + \mathbf{Zg} + \mathbf{Zg'} + \mathbf{e}$ to individual level data in females and males separately. The random effects vector was symbolically separated into two in the model to help model specification despite the same incidence matrix $\mathbf{Z}$ with rows of unit vectors where one component is 1 and all others are 0 indicating the respective line the individual was from. Furthermore, $\mathbf{W} = (1,\ldots,1)^T$; μ is the overall mean; the additive genetic value $\mathbf{g} \sim N(\mathbf{0}, \sigma_G{}^2\sigma^2_G\mathbf{G})$ is

assumed to be multivariate normal where **G** is the genomic relationship matrix of the n lines (also see below); the non-additive genetic value $\mathbf{g'} \sim N(\mathbf{0}, \sigma_G{}^2\sigma^2_{\mathrm{G}}\mathbf{I})$ where **I** is the identity matrix; and the error term $\mathbf{e} \sim N(\mathbf{0}, \sigma_E{}^2\mathbf{I})$. All variance components were estimated by REML using the Proc Mixed procedure in SAS software (Version 9.2 for Linux, [46]).

Genome wide association analysis

We performed genome wide association analysis on line means. Associations between single variants and line means for chill coma recovery time were tested by a mixed effect model accounting for relatedness among lines (including relatedness due to shared inversion karyotopes) using FastLMM [18, 47]. We used models of form $Y = \mu + V + a + \varepsilon$, where V is the fixed effect of the polymorphic variant and a is a polygenic term whose covariance is specified by the genomic relationship matrix to evaluate the effects of markers for males and females separately. We performed these analyses for 1,865,879 biallelic variants for which the Phred scaled variant quality ($10\log_{10}P$ where P is the probability value from a likelihood ratio test testing the existence of a variant) was greater than 500, the minor allele frequency was ≥ 0.05, and genotype call rate was ≥ 0.8 among the DGRP lines [18]. We considered only homozygous genotypes whose JGIL [48] quality scores were greater than 20. We estimated marginal allelic effects of each variant as one-half the difference in trait mean between the variant classes (polarized by allele frequency, such that the effect is the difference between the major and minor alleles) [25].

Bioinformatics analyses

We used SnpEff [49] for functional annotation of DNA variants based on the 5.49 release of the FlyBase [50] annotation.

Genomic Best Linear Unbiased Prediction (GBLUP)

We used the underlying statistical model $\mathbf{y} = \mathbf{W}\mu + \mathbf{Zg} + \mathbf{e}$ (Model 1) to perform genomic prediction. The ith component of the q-vector **y** is the phenotypic value of the ith line that is used for prediction (i = 1,. . .,n), $\mathbf{W} = (1,. . .,1)^T$; μ is the overall mean; $\mathbf{g} \sim N(\mathbf{0}, \sigma_G{}^2\sigma^2_{\mathrm{G}}\mathbf{G})$ is assumed to be multivariate normal where **G** is a genomic relationship matrix of the n lines; **Z** is a $(q\times n)$ incidence matrix with rows of unit vectors where one component is 1 and all others are 0, indicating the respective positions of lines used for prediction in the **g** vector of genetic values of all lines; $\sigma_G{}^2$ is the genetic variance; and $\mathbf{e} \sim N(\mathbf{0}, \sigma_E{}^2\mathbf{I})$ is the residual term, where $\sigma_E{}^2$ is the residual variance. The BLUP of the vector of genetic values can be obtained by solving the mixed model equations

$$\begin{pmatrix} \mathbf{W}^{\mathrm{T}}\mathbf{W} & \mathbf{W}^{\mathrm{T}}\mathbf{Z} \\ \mathbf{Z}^{\mathrm{T}}\mathbf{W} & \mathbf{Z}^{\mathrm{T}}\mathbf{Z} + \frac{\sigma_E^2}{\sigma_G^2}\mathbf{G}^{-1} \end{pmatrix} \begin{pmatrix} \hat{\mu} \\ \hat{\mathbf{g}} \end{pmatrix} = \begin{pmatrix} \mathbf{W}^{\mathrm{T}}\mathbf{y} \\ \mathbf{Z}^{\mathrm{T}}\mathbf{y} \end{pmatrix}.$$

When including two different random components, we used the statistical model $\mathbf{y} = \mathbf{W}\mu + \mathbf{Z_1 g_1} + \mathbf{Z_2 g_2} + \mathbf{e}$ (Model 2) where $\mathbf{g_1} \sim \mathrm{N}(0, \sigma_{G1}{}^2\mathbf{G_1})$ and $\mathbf{g_2} \sim \mathrm{N}(0, \sigma_{G2}{}^2\mathbf{G_2})$ are assumed to be independent multivariate normal vectors where $\mathbf{G_1}$ and $\mathbf{G_2}$ are two different genomic relationship matrices, and $\mathbf{Z_1}$ and $\mathbf{Z_2}$ are the corresponding incidence matrices. The BLUP for this

model can be obtained by solving the mixed model equations

$$\begin{pmatrix} \mathbf{W}^{\mathrm{T}}\mathbf{W} & \mathbf{W}^{\mathrm{T}}\mathbf{Z}_1 & \mathbf{W}^{\mathrm{T}}\mathbf{Z}_2 \\ \mathbf{Z}_1^{\mathrm{T}}\mathbf{W} & \mathbf{Z}_1^{\mathrm{T}}\mathbf{Z}_1 + \frac{\sigma_E^2}{\sigma_{G1}^2}\mathbf{G}_1^{-1} & \mathbf{Z}_1^{\mathrm{T}}\mathbf{Z}_2 \\ \mathbf{Z}_2^{\mathrm{T}}\mathbf{W} & \mathbf{Z}_2^{\mathrm{T}}\mathbf{Z}_1 & \mathbf{Z}_2^{\mathrm{T}}\mathbf{Z}_2 + \frac{\sigma_E^2}{\sigma_{G2}^2}\mathbf{G}_2^{-1} \end{pmatrix} \begin{pmatrix} \hat{\mu} \\ \widehat{\mathbf{g}_1} \\ \widehat{\mathbf{g}_2} \end{pmatrix} = \begin{pmatrix} \mathbf{W}^{\mathrm{T}}\mathbf{y} \\ \mathbf{Z}_1^{\mathrm{T}}\mathbf{y} \\ \mathbf{Z}_2^{\mathrm{T}}\mathbf{y} \end{pmatrix}.$$

We estimated variance components for Model 1 and Model 2 using maximum likelihood (ML) as implemented by the R package"RandomFields", version 2.0.46, and the "fitvario" function (http://CRAN.R-project.org/package=RandomFields) [51].

Genomic relationship matrices

We constructed genomic relationship matrices from the 3,742,106 SNPs and 437,096 indels that were polymorphic in the 176 DGRP lines, as well as for the subset of 1,868,905 common variants (MAF ≥ 0.05). We only used variants with a call rate > 0.8 in the 176 lines. Missing genotypes were assigned the allele frequencies based on the set of 176 lines.

The additive genomic relationship matrix, **G**, is defined as $\mathbf{G} = \frac{(\mathbf{M}-\mathbf{P})(\mathbf{M}-\mathbf{P})^{\mathrm{T}}}{2\sum_{j=1}^{s} p_j(1-p_j)}$ [5], where **M** is the (n×s) matrix of genotype vectors for the n lines, with the s variants coded as -1, 1; and the jth column of **P** is $(2(p_j—0.5),...,2(p_j - 0.5))^T$, where p_j is the frequency of the second allele at locus j.

We calculated pairwise epistatic genomic relationship matrices by modifying an approach according to Astle and Balding [23]. For the j-th pair ($\mathrm{SNP_{j1}}$, $\mathrm{SNP_{j2}}$) of interacting SNPs we built two relationship matrices, $\mathrm{K_{j1}}$ and $\mathrm{K_{j2}}$ (one for each of the two SNPs), according to

$$\mathbf{K}_{\mathbf{ji}} = \frac{(\mathbf{x}_{ji} - 2p_{ji}\mathbf{1})(\mathbf{x}_{ji} - 2p_{ji}\mathbf{1})^{\mathrm{T}}}{2p_{ji}(1 - p_{ji})}, \; i = 1, 2,$$

where $\mathbf{x}_{ji}$ is the genotype-vector and p_{ji} is the allele frequency of $\mathrm{SNP_{ji}}$ for i = 1, 2. We then calculated the Hadamard-product of $\mathbf{K_{j1}}$ and $\mathbf{K_{j2}}$ to obtain a matrix reflecting the interaction of $\mathrm{SNP_1}$ and $\mathrm{SNP_2}$. This calculation was repeated for all pairs of interacting SNPs and finally averaged over all Hadamard-products, to obtain one final epistatic relationship matrix

$$K = \frac{1}{m}\sum\nolimits_{j=1}^{m} K_{j1} \# K_{j2}$$

to be used as the pairwise epistatic genomic relationship matrix in the GBLUP models. In the additive case, the resulting covariance matrix is an unbiased and positive semi-definite estimator for the relationship matrix [23], and analogously K is an unbiased and positive semi-definite estimator for the additive x additive relationship matrix for the respective subset of m SNP pairs.

Calculating the epistatic genomic relationship matrix as the Hadamard product of the additive genomic relationship matrices constructed from all SNPs involved in epistatic interactions [13] accounts for all pairwise interactions of the involved SNPs. Thus, for n epistatic SNP pairs comprising $2n$ SNPs there are $4n^2$ SNP by SNP interactions. Our approach accounts for only the n pairwise interactions, *i.e.*, for a SNP interacting with one other SNP, just this interaction is modeled and the interactions with the SNPs in the other $n - 1$ epistatic pairs are disregarded. In more general terms it is always possible to split the set of all pairwise SNP interactions in two subsets, where one subset reflects all interactions with a significant effect, and the

complementary subset comprises all other pairwise interactions. The approach we used is equivalent to accounting for the significant interactions and ignoring the other interactions.

5-fold cross-validation using GBLUP

We first used 5-fold cross-validation (CV) [52–54] to assess prediction accuracy. In a 5-fold CV, the lines are randomly divided into five groups. Four of the five groups comprise the training set, and the remaining group constitutes the validation set, giving rise to five possible divisions of training and validation sets. For each of these divisions ("folds"), total genetic values for the lines of the validation set are predicted and the corresponding predictive ability defined as the correlation between predicted genetic values and observed phenotypic values is calculated. The five predictive abilities are then averaged to obtain one average correlation per CV replicate. These analyses were performed separately for males and females, with 100 replicates for each CV.

Leave-One-Out CV

We also performed a leave-one-out cross-validation (LOOCV) for trait-associated GBLUP. With n lines, the LOOCV consists of n folds. In each fold, n-1 observations are used as the training set and the phenotype of the remaining single line is predicted. This is repeated n times such that each line is predicted once. In each of the 176 folds, a GWA analysis for single variants and/or pair-wise interactions between variants was performed on the 175 lines of the training set only, *i.e.*, all single marker and epistatic GWA analyses were repeated 176 times. The single marker GWA analysis was performed as described above while the epistatic GWA was performed as follows. We first pruned the genotype data for LD using the LD pruning utility in PLINK [55] such that no pair of variants has $r^2 > 0.8$ within a window of 100 variants, and constrained the analysis to interactions between variants of MAF > 0.15 and at least two lines for each of the four possible genotypes. Raw phenotypic data were adjusted for the effects of inversions and major principal components of the genotypic matrix for common variants by fitting them as fixed effects and taking residuals from the fitted model [18]. Finally we performed a full genome-wide screen for pairwise interactions, fitting models of form $Y = \mu + V_A + V_B + V_A \times V_B + \varepsilon$, using FastEpistasis [56]. After the single marker and epistatic GWA analyses, we selected the top trait-associated additive variants and/or epistatic pairs with $p < 10^{-X}$ in the respective training set to construct genomic relationship matrices and predicted the phenotype of the remaining line based on Model 1 and Model 2, incorporating additive and/or epistatic genomic relationship matrices as described above. We computed the predictive ability as the correlation between the vector of estimated genetic values (from all 176 folds) and the vector of observed line phenotypes, and varied the P-value threshold X to arrive at an optimal value. The same LOOCV approach was also applied using the bigRR package, which implements a variant ridge regression with variant specific shrinkage parameters [30]. For comparison with the trait-associated GBLUP, we also performed LOOCV with two variance components, the additive genomic relationship matrix, **G**, and the Hadamard product of **G**, **G#G**, with the latter of the two representing genome-wide pair-wise interactions among all variants. All cross-validation procedures and the GBLUP approach were implemented using R software [51].

Simulations

To investigate whether the genetic architecture of a quantitative trait could account for the difficulty of the additive genomic relationship to explain phenotypic variation, we performed simulations under three distinct genetic architectures: (1) a major gene explaining 37% of the

phenotypic variation; (2) 100 loci additively explaining 37% of the phenotypic variation; and (3) 50 pairs of interacting loci explaining a total of 37% of the phenotypic variation. For the major gene and polygenic models, we randomly selected QTL sites from the genome and assigned their allelic effects such that each locus explained an equal amount of variance. For each pair of the randomly chosen QTLs in the epistatic model, the genotypic effect was assigned by the formula $b(m_1 - p_1)(m_2 - p_2)$, where m_1 and m_2 were the {-1, 1} coded genotypes, p_1 and p_2 were the allele frequencies of the two interacting loci, and b was the epistatic effect. To achieve equal variance for each interacting pair, we first calculated the sample variance of $(m_1 - p_1)(m_2 - p_2)$ and determined b accordingly.

Supporting Information

S1 Fig. Prediction accuracy using ridge regression (bigRR) and in the presence of genome-wide pair-wise epistatic variance. We performed LOOCV in females (**A**, **B**) and males (**C**, **D**) separately using a ridge regression approach implemented in the bigRR package [29] (**A**, females; **C**, males) and in the presence of genome-wide pair-wise epistatic variance (**B**, females; **D**, males).
(TIF)

S1 Table. Chill coma data for 176 DGRP lines. (A) Raw data. (B) Line means.
(XLSX)

S2 Table. Analysis of variance of chill coma recovery time using individual trait data.
(DOCX)

S3 Table. Top variants ($P < 10^{-5}$) associated with of chill coma recovery time in females and males.
(XLSX)

S4 Table. Variance components from models partitioning total genetic and additive genetic variance using the genomic relationship matrix.
(DOCX)

S5 Table. Stability analysis of additive variants in LOOCV GWAS for chill coma recovery time in males and females.
(XLSX)

S6 Table. Stability analysis of pairwise interactions in LOOCV GWAS for chill coma recovery time in males and females.
(XLSX)

Author Contributions

Conceived and designed the experiments: UO WH MM MS HS TFCM. Performed the experiments: UO WH MM. Analyzed the data: UO WH MM HS. Wrote the paper: UO WH HS TFCM.

References

1. Mackay TFC, Stone EA, Ayroles JF. The genetics of quantitative traits: Challenges and prospects. Nat Rev Genet. 2009; 10: 565–577. doi: 10.1038/nrg2612 PMID: 19584810
2. Visscher PM, Brown MA, McCarthy MI, Yang J. Five years of GWAS discovery. Am J Hum Genet. 2012; 90: 7–24. doi: 10.1016/j.ajhg.2011.11.029 PMID: 22243964

3. Manolio TA, Collins FS, Cox NJ, Goldstein DB, Hindorff LA, Hunter DJ, et al. Finding the missing heritability of complex diseases. Nature 2009; 461: 747–753. doi: 10.1038/nature08494 PMID: 19812666
4. Meuwissen THE, Hayes BJ, Goddard ME. Prediction of total genetic value using genomewide dense marker maps. Genetics 2001; 157: 1819–1829. PMID: 11290733
5. VanRaden PM. Efficient methods to compute genomic predictions. J Dairy Sci. 2008; 91: 4414–4423. doi: 10.3168/jds.2007-0980 PMID: 18946147
6. Yang J, Benyamin B, McEvoy BP, Gordon S, Henders AK, Nyholt DR, et al. Common SNPs explain a large proportion of the heritability for human height. Nat Genet. 2010; 42: 565–569. doi: 10.1038/ng.608 PMID: 20562875
7. Yang J, Manolio TA, Pasquale LR, Boerwinkle E, Caporaso N, Cunningham JM, et al. Genome partitioning of genetic variation for complex traits using common SNPs. Nat Genet. 2011; 43: 519–525. doi: 10.1038/ng.823 PMID: 21552263
8. Schaeffer LR. Strategy for applying genome-wide selection in dairy cattle. J Anim Breed Genet. 2006; 123: 218–223. PMID: 16882088
9. Goddard M. Genomic selection: prediction of accuracy and maximisation of long-term response. Genetica 2009; 185: 1021–1031.
10. Hayes BJ, Bowman PJ, Chamberlain AJ, Goddard ME. Genomic selection in dairy cattle: Progress and challenges. J Dairy Sci. 2009; 92: 433–443. doi: 10.3168/jds.2008-1646 PMID: 19164653
11. Lorenz AJ, Chao S, Asoro FG, Heffner EL, Hayashi T, Iwata H, et al. Genomic selection in plant breeding: Knowledge and prospects. Adv Agron. 2011; 110: 77–123.
12. Hayes BJ, Lewin HA, Goddard ME. The future of livestock breeding: genomic selection for efficiency, reduced emissions intensity, and adaptation. Trends Genet. 2013; 29: 206–214. doi: 10.1016/j.tig.2012.11.009 PMID: 23261029
13. Ober U, Ayroles JF, Stone EA, Richards S, Zhu D, Stricker C, et al. Using whole-genome sequence data to predict quantitative trait phenotypes in *Drosophila melanogaster*. PLoS Genet. 2012; 8: e1002685. doi: 10.1371/journal.pgen.1002685 PMID: 22570636
14. de Los Campos G, Vazquez AI, Fernando R, Klimentidis YC, Sorensen D. Prediction of complex human traits using the genomic best linear unbiased predictor. PLoS Genet. 2013; 9: e1003608. doi: 10.1371/journal.pgen.1003608 PMID: 23874214
15. Hu Z, Li Y, Song X, Han Y, Cai X, Xu S, et al. Genomic value prediction for quantitative traits under the epistatic model. BMC Genetics 2011; 12: 15. doi: 10.1186/1471-2156-12-15 PMID: 21269439
16. Long N, Gianola D, Rosa G, Weigel KA. Marker-assisted prediction of non-additive genetic values. Genetica 2011; 139: 843–845. doi: 10.1007/s10709-011-9588-7 PMID: 21674154
17. Mackay TFC, Richards S, Stone EA, Barbadilla A, Ayroles JF, Casillas S, et al. The *Drosophila melanogaster* Genetics Reference Panel. Nature 2012; 482: 173–178. doi: 10.1038/nature10811 PMID: 22318601
18. Huang W, Massouras A, Inoue Y, Peiffer J, Ràmia M, Tarone AM, et al. Natural variation in genome architecture among 205 *Drosophila melanogaster* Genetic Reference Panel lines. Genome Res. 2014; 24: 1193–1208. doi: 10.1101/gr.171546.113 PMID: 24714809
19. Anderson AR, Hoffmann AA, McKechnie SW. Response to selection for rapid chill-coma recovery in *Drosophila melanogaster*: physiology and life-history traits. Genet Res. 2005; 85: 15–22. PMID: 16089033
20. Macmillan HA, Sinclair BJ. Mechanisms underlying insect chill-coma. J Insect Physiol. 2011; 57: 12–20. doi: 10.1016/j.jinsphys.2010.10.004 PMID: 20969872
21. Huang W, Richards S, Carbone MA, Zhu D, Anholt RRH, Ayroles JF, et al. Epistasis dominates the genetic architecture of *Drosophila* quantitative traits. Proc Natl Acad Sci USA 2012; 109: 15553–15559. PMID: 22949659
22. de los Campos G, Sorensen D, Gianola D. Genomic heritability: What is it? Proceeding of the 10th World Congress of Genetics Applied to Livestock Production. 2014. Available: https://asas.org/wcgalp-proceedings
23. Astle W, Balding DJ. Population structure and cryptic relatedness in genetic association studies. Statist Sci. 2009; 24: 451–471.
24. Flint J, Mackay TFC. Genetic architecture of quantitative traits in mice, flies, and humans. Genome Res. 2009; 19: 723–733. doi: 10.1101/gr.086660.108 PMID: 19411597
25. Falconer DS, Mackay TFC. Introduction to Quantitative Genetics. 4th ed. Harlow: Longman Group Limited; 1996.

26. Daetwyler HD, Pong-Wong R, Villanueva B, Woolliams JA. The impact of genetic architecture on genome-wide evaluation methods. Genetics 2010; 185: 1021–1031. doi: 10.1534/genetics.110.116855 PMID: 20407128
27. Daetwyler HD, Calus MP, Pong-Wong R, de Los Campos G, Hickey JM. Genomic prediction in animals and plants: simulation of data, validation, reporting, and benchmarking. Genetics 2013; 193: 347–365. doi: 10.1534/genetics.112.147983 PMID: 23222650
28. de los Campos G, Hickey JM, Pong-Wong R, Daetwyler HD, Calus MP. Whole-genome regression and prediction methods applied to plant and animal breeding. Genetics 2013; 193: 327–345. doi: 10.1534/genetics.112.143313 PMID: 22745228
29. Gianola D. Priors in whole-genome regression: the bayesian alphabet returns. Genetics 2013; 194: 573–596. doi: 10.1534/genetics.113.151753 PMID: 23636739
30. Shen X, Alam M, Fikse F, Ronnegard L. A novel generalized ridge regression method for quantitative genetics. Genetics 2013; 193: 1255–1268. doi: 10.1534/genetics.112.146720 PMID: 23335338
31. Makowsky R, Pajewski NM, Klimentidis YC, Vazquez AI, Duarte CW, Allison DB, et al. Beyond missing heritability: Prediction of complex traits. PLoS Genet. 2011, 7: e1002051. doi: 10.1371/journal.pgen.1002051 PMID: 21552331
32. Zhang Z, Liu J, Ding X, Bijma P, de Koning DJ, Zhang Q. Best linear unbiased prediction of genomic breeding values using a trait-specific marker-derived relationship matrix. PLoS One 2010; 5: e12648. doi: 10.1371/journal.pone.0012648 PMID: 20844593
33. Hill WG, Goddard ME, Visscher PM. Data and theory point to mainly additive genetic variance for complex traits. PLoS Genet. 2008; 4: e1000008. doi: 10.1371/journal.pgen.1000008 PMID: 18454194
34. Mackay TFC. Epistasis and quantitative traits: using model organisms to study gene-gene interactions. Nat Rev Genet. 2014; 15: 22–33. doi: 10.1038/nrg3627 PMID: 24296533
35. Carlborg O, Jacobsson L, Ahgren P, Siegel P, Andersson L. Epistasis and the release of genetic variation during long-term selection. Nat Genet. 2006; 38: 418–420. PMID: 16532011
36. Kroymann J, Mitchell-Olds T. Epistasis and balanced polymorphism influencing complex trait variation. Nature 2005; 435: 95–98. PMID: 15875023
37. Steinmetz LM, Sinha H, Richards DR, Spiegelman JI, Oefner PJ, McCusker JH, et al. Dissecting the architecture of a quantitative trait locus in yeast. Nature 2002; 416: 326–330. PMID: 11907579
38. Sinha H, David L, Pascon RC, Clauder-Münster S, Krishnakumar S, Nguyen M, et al. Sequential elimination of major-effect contributors identifies additional quantitative trait loci conditioning high-temperature growth in yeast. Genetics 2008; 180: 1661–1670. doi: 10.1534/genetics.108.092932 PMID: 18780730
39. Stam LF, Laurie CC. Molecular dissection of a major gene effect on a quantitative trait: The level of alcohol dehydrogenase expression in *Drosophila melanogaster*. Genetics 1996; 144: 1559–1564. PMID: 8978044
40. Waddington CH. Canalization of development and genetic assimilation of acquired characters. Nature 1959; 183: 1654–1655. PMID: 13666847
41. Greene CS, Penrod NM, Williams SM, Moore JH. Failure to replicate a genetic association may provide important clues about genetic architecture. PLoS One 2009; 4: e5639. doi: 10.1371/journal.pone.0005639 PMID: 19503614
42. Zuk O, Hechter E, Sunyaev SR, Lander ES. The mystery of missing heritability: Genetic interactions create phantom heritability. Proc Natl Acad Sci USA 2012; 109: 1193–1198. doi: 10.1073/pnas.1119675109 PMID: 22223662
43. Kauffman SA. The Origins of Order. Oxford: Oxford University Press; 1993.
44. Phillips PC. Epistasis–the essential role of gene interactions in the structure and evolution of genetic systems. Nat Rev Genet. 2008; 9: 855–867. doi: 10.1038/nrg2452 PMID: 18852697
45. Morgan TJ, Mackay TFC. Quantitative trait loci for thermotolerance phenotypes in *Drosophila melanogaster*. Heredity 2006; 96: 232–242. PMID: 16404413
46. SAS Institute Inc. SAS 9.2 Help and Documentation. Cary: Statistical Analysis Software; 2013.
47. Lippert C, Listgarten J, Liu Y, Kadie CM, Davidson RI, Heckerman D. FaST linear mixed models for genome-wide association studies. Nat Methods 2011; 8: 833–835. doi: 10.1038/nmeth.1681 PMID: 21892150
48. Stone EA. Joint genotyping on the fly: identifying variation among a sequenced panel of inbred lines. Genome Res. 2012; 22: 966–974. doi: 10.1101/gr.129122.111 PMID: 22367192
49. Cingolani P, Platts A, Coon M, Nguyen T, Wang L, Wang L, et al. A program for annotating and predicting the effects of single nucleotide polymorphisms, SnpEff: SNPs in the genome of *Drosophila melanogaster strain* w^{1118}; iso-2; iso-3. Fly 2012; 6: 80–92. doi: 10.4161/fly.19695 PMID: 22728672

50. McQuilton P, St. Pierre SE, Thurmond J, The FlyBase Consortium. FlyBase 101 –the basics of navigating FlyBase. Nucleic Acids Res. 2012; 40: D706–714. doi: 10.1093/nar/gkr1030 PMID: 22127867

51. R Core Team. R: A language and environment for statistical computing. 2013; R Foundation for Statistical Computing. Available: http://www.R-project.org/. doi: 10.3758/s13428-013-0330-5 PMID: 23519455

52. Stone M. Cross-validation choice and assessment of statistical predictions. J Roy Statist Soc B. 1974; 36: 111–147.

53. Stone M. An asymptotic equivalence of choice of model by cross-validation and Akaike's criterion. J Roy Statist Soc B. 1977; 39: 44–47.

54. Allen D. The relationship between variable selection and data augmentation and a method of prediction. Technometrics 1977; 16: 125–127.

55. Purcell S, Neale B, Todd-Brown K, Thomas L, Ferreira MA, Bender D, et al. PLINK: a tool set for whole-genome association and population-based linkage analyses. Am J Hum Genet. 2007; 81: 559–575. PMID: 17701901

56. Schüpbach T, Xenarios I, Bergmann S, Kapur K. FastEpistasis: a high performance computing solution for quantitative trait epistasis. Bioinformatics 2010; 26: 1468–1469. doi: 10.1093/bioinformatics/btq147 PMID: 20375113
